# Prognostic Factors in Merkel Cell Carcinoma: A Retrospective Single-Center Study in 90 Patients

**DOI:** 10.3390/cancers10100350

**Published:** 2018-09-24

**Authors:** Marco Rastrelli, Beatrice Ferrazzi, Francesco Cavallin, Vanna Chiarion Sileni, Jacopo Pigozzo, Alessio Fabozzi, Saveria Tropea, Antonella Vecchiato, Alessandra Costa, Alessandro Parisi, Carlo Riccardo Rossi, Paolo Del Fiore, Mauro Alaibac

**Affiliations:** 1 Surgical Oncology Unit, Veneto Institute of Oncology IOV–IRCCS, 35121 Padua, Italy; marco.rastrelli@iov.veneto.it (M.R.); saveria.tropea@iov.veneto.it (S.T.); antonella.vecchiato@iov.veneto.it (A.V.); alessandra.costa@iov.veneto.it (A.C.); paolo.delfiore@iov.veneto.it (P.D.F.); 2 Dermatology Unit and Pathology Unit, Department of Medicine (DIMED), University of Padua, 35121 Padua, Italy; ferrazzi.beatrice@gmail.com; 3 Independent Statistician, 35121 Solagna, Italy; cescocava@libero.it; 4 Melanoma and Esophagus Oncology Unit, Veneto Institute of Oncology IOV-IRCCS, 35121 Padua, Italy; vanna.chiarion@iov.veneto.it (V.C.S.); jacopo.pigozzo@iov.veneto.it (J.P.); alessio.fabozzi@iov.veneto.it (A.F.); 5 Radiotherapy Unit, Veneto Institute of Oncology, IOV–IRCCS, 35121 Padua, Italy; alessandro.parisi@iov.veneto.it; 6 Department of Surgery, Oncology and Gastroenterology, University of Padua, 35121 Padua, Italy; carlor.rossi@unipd.it; 7 Dermatology Unit, Department of Medicine (DIMED), University of Padova, 35128 Padua, Italy

**Keywords:** Merkel cell carcinoma, neuroendocrine neoplasm, skin neoplasm, survival, CK20 expression, immunomodulatory drugs

## Abstract

Merkel Cell Carcinoma (MCC) is a rare but highly aggressive neuroendocrine neoplasm of the skin. This study aimed at describing characteristics, treatment, and prognosis of a series of consecutive cases of MCC patients, in order to contribute to the investigation of this rare malignancy and provide better patient care. This is a retrospective cohort study including all 90 patients diagnosed and/or treated for MCC between 1991 and 2018 at the Veneto Institute of Oncology in Padua (Italy). Patient and tumor characteristics, treatment, and immunohistochemical data were extracted from a prospectively collected local database. There were 68 primary (76%) and 22 non-primary (15 occult primary, three metastatic, four recurrence) tumors (24%). CK20 expression was associated with reduced overall (HR 2.92, 95% CI 1.04–8.16) and disease-specific (HR 4.62, 95% CI 1.31–16.28) survival. Immunomodulatory regimens for treatment of other comorbidities were associated with reduced disease-specific ((HR 2.15, 95% CI 1.06–4.36) and recurrence-free (HR 3.08, 95% CI 1.44–6.57) survival. Iatrogenic immunomodulation resulted as the main factor associated with impaired prognosis. Lack of CK20 expression was associated with better survival.

## 1. Introduction

**Figure 1:**
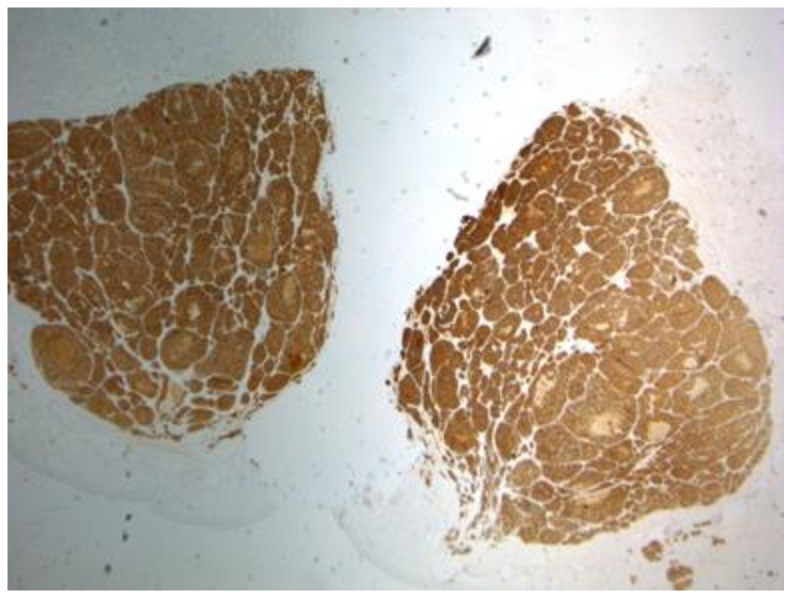
CK20 staining of a case of MMC.

Merkel Cell Carcinoma (MCC) is a rare neuroendocrine neoplasm of the skin [1]. It was first described by Toker in 1972 as trabecular carcinoma of the skin [2] and then renamed in its current form because of common features with Merkel cells [3]. Consequently, Merkel cells were suggested to be the source of MCC, on the basis of both the ultrastructural finding of neuroendocrine granules and the immunohistochemical expression of CK20 (Figure 1) and CD56 [4,5,6]. However, the exact tumor genesis is still unclear [7,8].

The incidence rate of MCC varies across different regions of the world (from 0.13 in Europe to 1.6 per 100,000 in Australia) [9]. MCC is usually asymptomatic with rapid expansion and preferential localization on head, neck and arms in skin exposed to sunlight [3,9]. Risk factors include older age, immunosuppression, exposure to ultraviolet (UV) radiation, fair skin, and previous malignancies [1,9]. The immune system has an essential role in preventing the development of MCC, as suggested by the highest incidence of MCC in patients with hematological malignancies, HIV infection, organ transplantation, and in those treated with immunosuppressive agents [10,11,12].

Because of the strong connection between the disease and immune suppression, a possible viral etiology of the disease was suspected. In 2008 Feng and Shuda, isolated an unknown polyomavirus in MCC’s tumor cells and named it Merkel Cell Polyomavirus (MCPyV). The virus is clonally integrated in 80% of MCCs, suggesting its oncogenetic role in the pathogenesis of the neoplasm [13]. On the basis of the discovery of MCPyV, two different subtypes of MCC can be individuated: MCC MCPyV+, which has a better prognosis, and MCPyV-, related to sun exposure, which has the highest mutation rate and more aggressive behavior [14].

MCC is highly aggressive and is the second most common cause of skin cancer death after melanoma [9]. Treatment strategy includes surgery (wide excision) in patients with locoregional primary MCC, followed by complete lymph node dissection in clinical node-positive patients. Sentinel node biopsy concurrent with wide excision is recommended in all patients with primary disease. Radiotherapy can be offered as adjuvant treatment after surgery and as primary or palliative treatment for inoperable cases, while chemotherapy is usually reserved for metastatic disease. Over the last few years immunotherapy has offered new therapeutic strategies and has been used for treatment of advanced MCC in patients not responsive to chemotherapy or as first-line treatment in metastatic disease. [9,15].

This study aimed at describing the characteristics, treatment, and prognosis of a series of 90 consecutive cases of MCC presented at our Institute.

## 2. Results

### 2.1. Patients

The study included 90 patients (50 male and 40 female; median age: 69 years) with a diagnosis of MCC observed between December 1991 and January 2018. Patient characteristics are shown in Table 1. There were 68 primary (76%) and 22 non-primary (15 occult primary, three metastatic, four recurrence) tumors (24%). MCC was generally observed on the extremities (63%), followed by the trunk/buttocks (19%) and head/neck (18%).

### 2.2. Treatment

**Figure 2:**
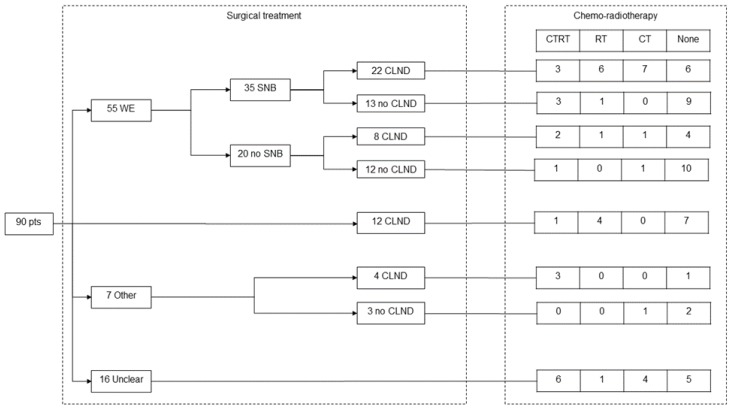
Surgical treatment and chemo-radiotherapy.

Surgical treatment and chemo-radiotherapy are shown in Figure 2. Primary surgical treatment was WE in 55 patients and CLND in 12. Seven patients underwent other surgical treatments (seven had wide resection), while the information was unclear in 16 patients who were treated in other hospitals. Following SNB, CLND identified median one positive lymph node (IQR 0–9). CLND was also performed in five patients with a negative SLN (median three positive lymph nodes, IQR 0-5). Among 46 patients who underwent CLND, extra capsular spread (ECS) was present in 20 (43%) patients and absent in 26 (57%) patients. Radiotherapy was administered to 32 (36%) patients (29 adjuvant, one neoadjuvant, two metastatic). Chemotherapy was administered to 33 (37%) patients (12 adjuvant, 21 metastatic).

### 2.3. Survival in Patients with Tumor Stage I–III

**Figure 3:**
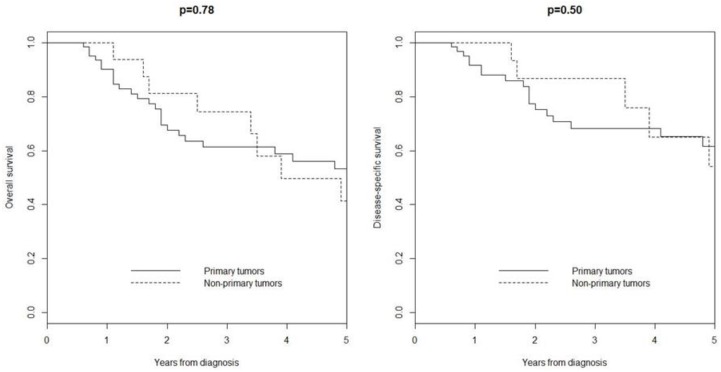
Overall survival and disease-specific survival in patients with tumor stage I–III.

Median follow-up was 29 months (IQR 13–61). Five-year overall survival was 53% in patients with primary tumor and 41% in those with non-primary tumors (*p* = 0.78), while the five-year disease-specific survival was 58% in patients with primary tumors and 54% in those with non-primary tumors (*p* = 0.50) (Figure 3). Univariate analyses of overall survival and disease-specific survival are shown in Table 2. Impaired overall survival was associated with age-adjusted Charlson comorbidity index (HR 1.22, 95% CI 1.01–1.49; *p* = 0.04) and CK20 expression (HR 2.82, 95% CI 1.01–7.93; *p* = 0.04). Impaired disease-specific survival was associated with CK20 expression (HR 4.46, 95% CI 1.26–15.82; *p* = 0.02).

SLN was positive in 15 patients and negative in 14 patients among those with primary tumor who received SNB concurrent with WE. Positive SLN seemed to be associated with impaired overall survival with respect to negative SLN (HR 4.49, 95% CI 0.87–23.27; *p* = 0.07). Disease-specific survival overlapped with overall survival.

In 35 patients with primary tumor who underwent CLND, a non-significant association of ECS with impaired overall survival was observed (HR 2.54, 95% CI 0.86–7.52; *p* = 0.09) and impaired disease-specific survival (HR 3.09, 95% CI 0.95–10.10; *p* = 0.06). In the same subgroup, having three or more positive LNs at CLND was not associated with overall survival (HR 1.66, 95% CI 0.59–4.71; *p* = 0.34) or disease-specific survival (HR 2.34, 95% CI 0.70–7.79; *p* = 0.17) with respect to having two or fewer positive LNs at CLND.

### 2.4. Recurrence among Patients with Primary Stage I–III MCC

**Figure 4:**
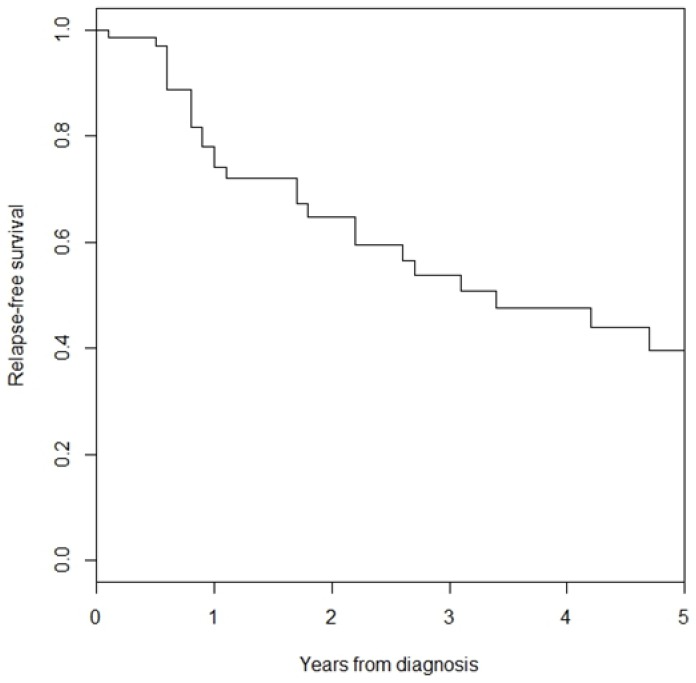
Recurrence -free survival among patients with primary stage I–III MCC.

At the time of the analysis, 28 patients with diagnosis of primary MCC developed disease recurrence. Local recurrence was observed in 16 patients, in-transit metastases in 6, LN metastases in 15 and distant metastases in 13. Five-year recurrence-free survival was 39% (Figure 4). Univariate analyses of recurrence-free survival are shown in Table 3. Impaired recurrence-free survival was associated with receiving immunomodulatory drugs (HR 2.72, 95% CI 1.22–6.10; *p* = 0.01) and radiotherapy (HR 2.72, 95% CI 1.28–5.77; *p* = 0.009).

Among the patients with primary tumor who received SNB concurrent with WE, positive SLN was not associated with recurrence-free survival (HR 1.11, 95% CI 0.35–3.58; *p* = 0.86).

Among the patients with primary tumor who underwent CLND, ECS was not associated with recurrence-free survival (HR 1.47, 95% CI 0.56–3.81; *p* = 0.43). In the same subgroup, having three or more positive LNs at CLND was not associated with recurrence-free survival (HR 1.88, 95% CI 0.68–5.18; *p* = 0.23) with respect to having two or fewer positive LNs at CLND.

### 2.5. Comparison of MCC with Occult Primary and Primary MCC with Positive LNs

Fifteen patients with MCC with occult primary were compared with 31 patients with primary MCC and positive LNs (Table 4). Tumor site was different between the two groups (*p* < 0.0001), with 73% of MCC with occult primary in trunk/buttocks and 77% of primary MCC with positive LNs in extremities (Table 4). Neoplastic comorbidity was present in eight patients (26%) with primary MCC and positive LNs, while it was absent in patients with MCC with occult primary (*p* = 0.04, Table 4). Excluding three patients with tumor stage IV, five-year overall survival was 58% in patients with MCC with occult primary and 47% in those with primary MCC and positive LNs (*p* = 0.18), while five-year disease-specific survival was 64% in patients with MCC with occult primary and 48% in those with primary MCC and positive LNs (*p* = 0.16).

## 3. Discussion

Our study describes patient characteristics, treatment strategy and prognosis of a series of 90 consecutive cases of MCC.

In our study, about one-fifth of patients received immunomodulatory drugs for the treatment of other comorbidities. Immunomodulatory drugs included treatments for transplanted patients and patients affected by autoimmune diseases (i.e., steroids or immunosuppressive drugs such as cyclosporin). In our study, an immunomodulatory regimen was associated with reduced DFS, in agreement with previous data [17]. The iatrogenic systemic immune suppression caused by immunomodulatory drugs could explain the higher risk of recurrence in these patients. Immunosuppressed MCC patients have been demonstrated to have a worsened prognosis [18]. Modifying immunosuppressive regimens to decrease the cumulative immunosuppressive load may provide some prognostic advantages in these patients.

In this study, the lack of CK20 expression in immunohistochemistry was associated with better survival (both overall and disease-specific), while the other immunohistochemical markers (NSE, synaptophysin and chromogranin) did not show any relationship with the prognosis. CK20 is a cytokeratin polypeptide that is usually expressed in the gastric and intestinal epithelium, urothelium, and Merkel cells [19], whereas NSE, synaptophysin, and chromogranin are endocrine-related markers [20]. Since the early 1990s, immunohistochemical staining of CK20 has been performed to support the histopathological diagnosis in MCC [20], but it has also been evaluated as prognostic factor in other malignancies [21]. Our findings may suggest a prognostic role for CK20 expression also in MCC, although this finding should be confirmed in further studies with a larger sample size.

Our data confirm the association between survival and both tumor stage and comorbidity status in agreement with previous studies [22,23,24]. Although tumor size larger than 2 cm and the number of positive lymph nodes have already been associated with a worse prognosis [25], the limited sample size may have prevented us from showing this association in our study. Unexpectedly, survival was not different between patients with primary MCC and those with non-primary MCC, which is not consistent with previous published data [22]. This finding could be due to the inclusion of occult primary tumors among non-primary MCC, since previous studies suggested a better prognosis in patients with occult MCC if compared to those with primary MCC with positive LNs [26]. In our study, MCC was generally observed on the extremities in primary MCC with positive LNs, and trunk/buttocks in occult MCC. Neoplastic comorbidity was present in 25% of patients with primary MCC with positive LNs, while it was absent in patients with MCC with occult primary. These findings could be explained by a more efficient immune surveillance in patients with occult primary.

In our study, positive SLN seemed to be associated with impaired survival in patients with primary tumor, thus suggesting an indication for complete LN dissection and/or radiotherapy. However, there are conflicting results about the role of sentinel lymph node status on patient survival [27,28]. In addition, ECS in patients with primary tumor receiving CLND seemed to be associated with reduced survival, as previously reported [29]. It is noteworthy that almost half patients who underwent CLND had ECS.

Disease recurrence occurred in a considerable number of patients with primary MCC during follow-up, in agreement with previous studies [22]. Immunomodulatory regimens were associated with reduced recurrence-free survival along with reduced disease-specific survival. The failed immune surveillance of the cancer following the iatrogenic systemic immune suppression is probably the main factor for disease recurrence [18]. Radiotherapy was also associated with reduced recurrence-free survival, probably because it is generally performed in patients with advanced disease [3].

Our data confirms previously reported patient features associated with the development of MCC, notably older age, CK20 expression, synaptophysin expression and chromogranin expression [9,19,20]. In addition, 20% of patients had previous neoplasm and about 25% of patients had autoimmune comorbidities [30]. Finally, our investigation supports the view that radiotherapy is associated with a better regional control in MCC [31].

Our findings are consistent with the important role of immunomodulatory drugs in prognosis, thus confirming the necessity of a multidisciplinary approach in patient assessment.

The present study has however some limitations. First, the retrospective nature of the study limited the availability of data (i.e., immunohistochemistry). Second, this study reported a single-center experience, thus the generalizability of the findings is limited to similar settings. Third, the investigation included patients who had been treated using various modalities because of the long period of inclusion and different stages at diagnosis. Finally, it was not possible, using the data available, to perform a multivariate analysis.

## 4. Materials and Methods

### 4.1. Study Design

This is a retrospective cohort study including all patients diagnosed and/or treated for MCC between December 1991 and January 2018 at the Melanoma and Sarcoma Clinic of the Veneto Institute of Oncology in Padua (Italy). These institutions are level III referral center located in Northeastern Italy. Most patients are referred for diagnosis and/or first-line treatment, while some patients are referred for disease progression after being treated in local level II centers. The ethical committee gave the ethical approval Not. 4/2018 on 23 July 2018. 

### 4.2. Diagnosis and Treatment

**Figure 5:**
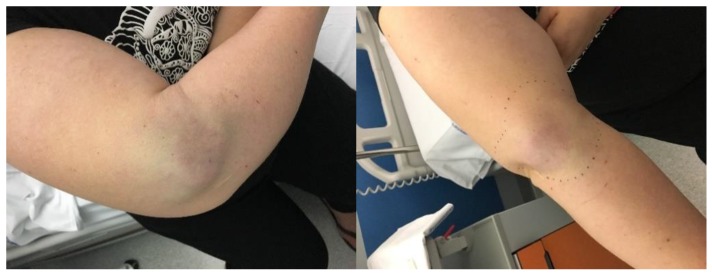
Patient with MCC. Lesion diameter 5 × 6 cm.

MCC was diagnosed according to histopathology and immunohistochemistry of the biopsy of the lesion. Tumor stage was defined according to the eighth version of the American Joint Committee on Cancer (AJCC) staging system, effective from January 2018 [16]. All diagnoses before January 2018 were re-staged according to the last version of the staging system. A clinical example of a MCC is shown in Figure 5. 

The surgical treatment included wide excision (WE) of the primary lesion, sentinel lymph node biopsy (SNB) and/or regional lymph nodes dissection. Patients with locoregional primary MCC underwent WE, followed by complete lymph node dissection in clinical node-positive patients. Sentinel node biopsy was performed concurrent with WE in patients with primary lesions. Patients with occult primary and clinical lymph node involvement received complete lymph node dissection (CLND). Immunotherapy and radio- and/or chemotherapy were offered based on tumor stage [15].

Radio-therapy was offered as adjuvant treatment in patients with operable disease, or as neo-adjuvant or palliative treatment for inoperable disease (i.e., recurrence or metastatic disease), while chemotherapy was usually reserved for metastatic disease. Immunotherapy has been used since 2017 in patients with metastatic disease who did not benefit from radio- and/or chemotherapy or as first line treatment in metastatic disease. Follow-up visits were performed every three to four months for the first three years, then every six months for up to five years and every year thereafter. A total-body CT scan was performed twice a year for the first five years. Disease progression included regional recurrences, in-transit metastases, lymph node metastases, and distant metastases.

### 4.3. Data Collection

All data were extracted from a prospectively collected local database. Demographics included age at diagnosis, sex and familiarity, while tumor information included presentation, size, anatomic location, and stage. Comorbidity status was summarized using age-adjusted Charlson Comorbidity Index. The age adjusted Charlson Comorbidity Index is a prognostic classification used for patients who may be affected by comorbid conditions. It takes into consideration 19 comorbidity categories and patient age and each condition is given a score depending on the risk of death because of this specific condition. The overall score is the sum of the weighted scores for all comorbidity conditions. Patients with higher score have greater comorbidity condition and/or older age and score >5 implies a 100% mortality risk over one year.

Neoplastic comorbidity and autoimmune comorbidity were evaluated separately. Autoimmune comorbidity could be organ-specific (i.e., autoimmune thyroiditis) or systemic (i.e., rheumatoid arthritis). We considered the immunomodulatory drugs used for the treatment of patients’ comorbidities such as long-term steroids, cyclosporine, and anti-rheumatic drugs. Immunohistochemistry data (the presence of CK20, NSE, Synaptophysin, and Chromogranin) were also retrieved. Information on treatment strategy included WE, SNB, CLND, immunotherapy, and radio- and/or chemotherapy. Follow-up information was extracted from scheduled visits. Overall survival was calculated from date of diagnosis to date of death or date of last visit. Disease-specific survival was calculated from date of diagnosis to date of disease-related death or date of last visit/disease-unrelated death. Recurrence-free survival was calculated in patients with primary MCC from date of diagnosis to date of recurrence or date of last visit/death.

### 4.4. Statistical Analysis

Continuous data were expressed as median and interquartile range (IQR). Categorical data were compared between two groups using Fisher’s exact test, while continuous data used Mann–Whitney test. Survival curves were calculated using the Kaplan–Meier method and compared with a log-rank test. The association between clinically relevant variables and survival (overall survival, disease-specific survival, and recurrence-free survival) was evaluated using Cox regression models, and expressed as the hazard ratio (HR) with a 95% confidence interval (95% CI). The association between survival and chemotherapy was not evaluated because chemotherapy was reserved for metastatic disease and thus was a proxy of metastatic disease rather than a possible risk factor for survival. The limited sample size and availability of immunohistochemistry data did not allow any meaningful multivariable analyses. All tests were two-sided and a *p*-value of less than 0.05 was considered statistically significant. Statistical analyses were performed using R 3.3 (R Foundation for Statistical Computing, Vienna, Austria) [32].

## 5. Conclusions

Our findings confirmed patient characteristics such as older age, CK20 expression, synaptophysin expression, and chromogranin expression. Previous neoplasm and autoimmune comorbidity were frequent. Immunosuppression was the main factor associated with impaired disease-free survival. The lack of CK20 expression in immunohistochemistry was associated with better survival. Our findings indicate the relevance of a multidisciplinary approach to patient assessment. Moreover, future multicenter trials are warranted, in particular with regard to the impact of the new immunotherapeutic approaches on overall survival in advanced stages [33].

## Figures and Tables

**Table 1:** Patient characteristics.

		All Patients	Non-Primary Tumors	Primary Tumors
	N Patients	90	22	68
Demographics	Age at diagnosis, years ^a^	69 (61–78)	69 (61–79)	66 (62–73)
	Sex:			
	Female	40 (44)	9 (41)	31 (46)
	Male	50 (56)	13 (59)	37 (54)
	Familiarity ^b^:			
	No	35 (39)	7 (32)	28 (41)
	Yes	13 (14)	4 (18)	9 (13)
	Information not available	42 (47)	11 (50)	31 (46)
Merkel cell Carcinoma	Tumor size:			
	≤2 cm	19 (21)	0	19 (28)
	>2 cm	71 (79)	22 (100)	49 (72)
	Anatomic location ^c^:			
	Head/neck	15 (20)	0 (0)	15 (22)
	Extremities	44 (59)	5 (71)	39 (57)
	Trunk/buttocks	16 (21)	2 (29)	14 (21)
	Tumor stage [16]:			
	I	19 (21)	0	19 (28)
	II	19 (21)	2 (9)	17 (25)
	III	47 (52)	17 (77)	30 (44)
	IV	5 (6)	3 (14)	2 (3)
Comorbidity	Age-adjusted Charlson comorbidity index ^a^	3 (2–4)	3 (2–4)	4 (2–5)
	Neoplastic comorbidity:			
	No	70 (78)	19 (86)	51 (75)
	Yes	20 (22)	3 (14)	17 (25)
	Autoimmune comorbidity:			
	No	66 (73)	14 (64)	52 (77)
	Organ-specific	7 (8)	2 (9)	5 (7)
	Systemic	13 (15)	4 (18)	9 (13)
	Both	4 (4)	2 (9)	2 (3)
Drugs	Immunomodulatory:			
	No	71 (81)	19 (86)	54 (79)
	Yes	17 (19)	3 (14)	14 (21)
	Statins: ^d^			
	No	81 (90)	19 (86)	62 (91)
	Yes	9 (10)	3 (14)	6 (9)
Immunohistochemistry	Immunohistochemistry availability, N patients	62	11	51
	CK20: expression	45 (73)	9 (82)	36 (71)
	NSE: expression	15 (24)	2 (18)	13 (25)
	Synaptophysin: expression	47 (76)	9 (82)	38 (75)
	Chromogranin: expression	41 (66)	6 (55)	35 (69)

Data expressed as n (%) or ^a^ median (IQR). ^b^ Familiarity: the patient had a parent and/or sister and/or brother affected by neoplasm. ^c^ information regarding 75 patients, patients with unknown primaries are excluded. ^d^ Patients receiving only statins for the treatment of other comorbidities.

**Table 2:** Univariate analysis of overall survival and disease-specific survival in patients with tumor stage I–III.

Patient Characteristics	Overall Survival		Disease-Specific Survival	
	HR (95% CI)	*p*-Value	HR (95% CI)	*p*-Value
Primary vs. non primary tumor	1.10 (0.54–2.30)	0.78	1.41 (0.53–3.742)	0.49
Age at diagnosis	1.02 (0.99–1.05)	0.17	1.01 (0.97–1.04)	0.62
Male vs. female	1.29 (0.68–2.45)	0.43	1.79 (0.79–4.02)	0.16
Anatomic location:				
Head/neck vs. extremities	1.34 (0.59–3.19)	0.46	1.99 (0.78–5.11)	0.15
Trunk/buttocks vs. extremities	1.22 (0.52–2.83)	0.64	1.23 (0.41–3.70)	0.71
Tumor size: >2 cm vs. ≤2 cm	1.60 (0.62–4.10)	0.33	1.37 (0.47–3.97)	0.57
Tumor stage: III vs. I–II	1.56 (0.82–2.95)	0.18	2.06 (0.91–4.69)	0.08
Age-adjusted Charlson comorbidity index	1.22 (1.00–1.49)	0.04	1.23 (0.97–1.56)	0.09
Neoplastic comorbidity: yes vs. no	0.79 (0.35–1.80)	0.58	0.92 (0.35–2.45)	0.87
Autoimmune comorbidity: yes vs. no	1.29 (0.60–2.76)	0.51	1.44 (0.60–3.46)	0.42
Immunomodulatory drugs: yes vs. no	1.13 (0.47–2.70)	0.79	1.42 (0.53–3.77)	0.49
CK20: expression vs. absence ^a^	2.82 (1.01–7.93)	0.04	4.46 (1.26–15.82)	0.02
NSE: expression vs. absence ^a^	0.60 (0.20–1.77)	0.35	0.71 (0.24–2.14)	0.55
Synaptophysin: expression vs. absence ^a^	2.19 (0.74–6.52)	0.16	1.84 (0.61–5.56)	0.28
Chromogranin: expression vs. absence ^a^	0.74 (0.31–1.75)	0.49	0.93 (0.35–2.44)	0.89
Radiotherapy: yes vs. no	0.87 (0.45–1.70)	0.68	1.61 (0.75–3.85)	0.22

^a^ Information available in 62 patients.

**Table 3:** Factors associated with recurrence-free survival among patients with primary stage I–III tumor.

Factors	Recurrence-Free Survival	
	HR (95% CI)	*p*-Value
Age at diagnosis	1.01 (0.98–1.05)	0.35
Male vs. female	0.81 (0.39–1.72)	0.59
Anatomic location:		
Head/neck vs. extremities	1.10 (0.41–2.94)	0.85
Trunk/buttocks vs. extremities	1.00 (0.13–7.53)	0.99
Tumor size: >2 cm vs. ≤2 cm	0.76 (0.33–1.74)	0.52
Tumor stage III vs. I–II	0.81 (0.38–1.73)	0.58
Age-adjusted Charlson comorbidity index	1.15 (0.94–1.42)	0.18
Neoplastic comorbidity: yes vs. no	0.85 (0.34–2.10)	0.72
Autoimmune comorbidity: yes vs. no	1.20 (0.48–2.97)	0.70
Immunomodulatory drugs: yes vs. no	2.72 (1.22–6.10)	0.01
CK20: expression vs. absence ^a^	1.43 (0.54–3.78)	0.48
NSE: expression vs. absence ^a^	0.68 (0.25–1.88)	0.46
Synaptophysin: expression vs. absence ^a^	1.96 (0.70–5.45)	0.20
Chromogranin: expression vs. absence ^a^	1.15 (0.44–2.99)	0.78
Radiotherapy: yes vs. no	2.72 (1.28–5.77)	0.009

^a^ Information available in 51 patients.

**Table 4:** Comparison of MCC with occult primary and primary MCC with positive LNs.

	MCC with Occult Primary	Primary MCC with Positive LNs	*p*-Value
N patients	15	31	-
Age at diagnosis, years ^a^	69 (61–72)	68 (58–76)	0.94
Sex:			0.52
Female	7 (47)	10 (32)	
Male	8 (53)	21 (68)	
Anatomic site:			<0.0001
Head/neck	1 (7)	4 (13)	
Extremities	3 (20)	24 (77)	
Trunk/buttocks	11 (73)	3 (10)	
Tumor stage:			0.99
III	14 (93)	29 (94)	
IV	1 (7)	2 (6)	
Age-adjusted Charlson comorbidity index ^a^	3 (2–3)	3 (2–5)	0.39
Neoplastic comorbidity:			0.04
No	15 (100)	23 (74)	
Yes	0	8 (26)	
Autoimmune comorbidity:			0.47
No	9 (60)	24 (77)	
Organ-specific	1 (7)	2 (7)	
Systemic	3 (20)	4 (13)	
Both	2 (13)	1 (3)	
Immunomodulatory drugs:			0.49
No	10 (67)	24 (77)	
Yes	5 (33)	7 (23)	
Immunohistochemistry availability, N patients	9	22	-
CK20	7 (78)	17 (77)	0.99
NSE	1 (11)	7 (32)	0.38
Synaptophysin	8 (89)	16 (73)	0.64
Chromogranin	5 (56)	16 (73)	0.42
Radiotherapy	7 (47)	15 (48)	0.99
Chemotherapy	5 (33)	14 (45)	0.53

Data expressed as n (%) or ^a^ median (IQR).

## Abbreviations

CI	confidence interval
CK	cytokeratin
CLND	complete lymph node dissection
ECS	extracapsular spread
HR	hazard ratio
LN	lymph node
MCC	Merkel cell carcinoma
SLN	sentinel lymph node
SNB	sentinel node biopsy
